# An antibiotic audit of the surgical department at a rural hospital in Western Kenya

**DOI:** 10.11604/pamj.2018.29.219.14510

**Published:** 2018-04-23

**Authors:** Ruth Chepkemoi Talaam, Michael Mudeheri Abungana, Philip Blasto Ooko

**Affiliations:** 1 Department of Surgery, AIC Litein Hospital, Litein, Kenya

**Keywords:** Antibiotic audit, irrational use, antibiotic resistance

## Abstract

**Introduction:**

Antibiotics are one of the most commonly prescribed medications in hospitalized patients, with up to half of prescriptions being irrational. This study aimed to assess the quality of antibiotic use among surgical inpatients at our institution.

**Methods:**

A one year (January 1-December 31, 2015) retrospective chart review on antibiotic use for patients admitted to the surgical department at AIC Litein Hospital, a faith based non-governmental health institution in Western Kenya, was conducted. Data were collected from medical and nursing patient charts with a standardized questionnaire. The criteria applied to assess inappropriate antibiotic use focused on the choice, duration and indication of the antibiotics prescribed.

**Results:**

A total of 394 cases were evaluated, with a mean age of 44.8 years and a mean duration of hospitalization of 7.2 days. Antibiotics were initiated either for prophylaxis (205, 56.3%) or treatment (159, 43.7%) for a mean duration of 6 days (range 1-37). The predominant route of administration was intravenous (332, 91.2%). Most antibiotics started at admission were continued till discharge and the duration of antibiotics was indicated in only 11% of the treatment sheets. At discharge, 321 (81.4%) cases had antibiotics prescribed for a mean duration of 5.7 days (range 1-60). Inappropriate prescriptions were noted in 45.4% of prophylactic antibiotics, 33.4% treatment antibiotics and 52.6% of discharge antibiotics. The most common reason for inappropriate antibiotic use during hospitalization was inappropriate duration (45.9%).

**Conclusion:**

Proper documentation, daily antibiotic review and preparation of a local antibiotic policy guideline could help improve the appropriate use of antibiotics.

## Introduction

Antibiotics are one of the most commonly prescribed medications in hospitalized patients, accounting for up to 50% of total drug cost [1-4]. Rational antibiotic use require that a safe, affordable, efficacious drug is given to the right patient, at the right time, at the right dose, for the right indication and for an appropriate duration [1,5,6]. A significant proportion (20-50%) of antibiotics prescribed in hospitals for prophylaxis or treatment has been reported to be irrational [1-3]. Absence of guidelines on rational antibiotic use, uncertainty over the differential diagnosis, presence of complex co-morbidities, wrong interpretation of microbial results and poor compliance to antibiotic protocols have all been implicated in inappropriate use of antibiotics [3,6,7]. This leads to increased costs for the patient, drug toxicity, poor patient outcomes and prolonged hospitalizations [2,6,7]. One of the most important factors driving the emergence of antibiotic resistance is the volume of drug use. As the quantity of drugs prescribed has risen; driven by frequent, incorrect and/or inappropriate duration of therapy, there has been a concurrent increase in the emergence of antibiotic resistance [2,3,5,8]. Infections caused by resistant organisms are associated with prolonged length of stay, higher morbidity and mortality, and higher overall costs compared to non-resistant organisms [9]. This study aimed to assess the quality of antibiotic prescription and reasons behind irrational antibiotic use at a single institution in Western Kenya.

## Methods

This retrospective review was undertaken at AIC Litein Hospital, a referral faith-based facility in Western Kenya, Kericho County, Litein town with 200-bed capacity, serving a population of up to 400,000 people. All in-patients admitted to the surgical department during the period of January 1^st^ to December 31^st^ 2015 were included in the study. Patients with incomplete/missing data were excluded from the study. Data concerning patient demographics, diagnosis, surgeries, name and duration of antibiotics prescribed, complications, outcome and duration of hospitalization were recorded from the individual case records. The data was then entered into a preformed data sheet and analyzed using descriptive statistics. An antibiotic given was considered to be inappropriate by the following criteria: 1) The antibiotics were prescribed for a condition that does not need treatment with antibiotics (inappropriate indication). 2) The antibiotic chosen was too broad, too narrow or ineffective for the particular condition (Inappropriate choice). 3) Wrong duration of therapy (Inappropriate duration). Permission to carry out this study was granted by the Institutional Research Board (IRB) at AIC Litein Hospital.

## Results

A total of 430 files were retrieved for analysis, with 36 being rejected due to inadequate information (n = 10), missing treatment sheets (n = 10) or admission to other wards (n = 16). The 394 cases represented 368 patients as 24 patients were admitted twice during the duration of the study, and one patient was admitted three times. The group had a mean age of 44.8 ± 22.2 years and a male predominance at 71.3%. The mean duration of hospitalization was 7.2 ± 6.2 days, with most patients (281, 71.3%) being admitted for 1-7 days. The rest were distributed as 78 (19.8%) for 8-14 days, 18 (4.6%) for 15-21 days and 17 (4.3%) for > 22 days. At discharge, 18 patients were referred for further management and while 7 patients died.

**Antibiotic use:** Antibiotics were initiated in 92% of cases, either for prophylaxis (205, 56.3%) or treatment (159, 43.7%). The mean duration administration was 6±4.7 days, with most patients being on antibiotics for 1-3 days (110, 30.2%) or 4-6 days (149, 40.9%). The antibiotics were prescribed at admission (251, 69%), in the ward (35, 9.6%) or post-operatively (78, 21.4%). Most patients received one (161, 44.2%) or two antibiotics (160, 44.2%), with only 43 (11.8%) patients receiving three or more antibiotics simultaneously. The mean number of antibiotics was 1.6 ± 0.6 per patient. The route of administration was intravenous in 332 (91.2%) cases and oral in 32 (8.8%) cases. Ceftriaxone and floxacillin, either alone or in combination with metronidazole, were the most common antibiotics prescribed (Table 1). While a total of 275 surgeries were undertaken in 240 patients, the use of preoperative antibiotics in theater was noted in only 127 (46.2%) cases.

**Table 1:** Most common antibiotics prescribed for inpatients during admission and at discharge at the surgical department between January and December 2015 at AIC Litein Hospital

Admission		Discharge	
Ceftriaxone	200 (32.3%)	Floxacillin	141 (39.3%)
Metronidazole	180 (29.1%)	Ampicillin-cloxacillin	64 (17.8%)
Floxacillin	135 (21.8%)	Metronidazole	50 (14%)
Penicilin G	19 (3.1%)	Cefuroxime	35 (9.8%)
Ampicillin-cloxacillin	16 (2.6%)	Amoxicillin-clavulanate	26 (7.3%)
Gentamycin	14 (2.3%)	Amoxicillin	11 (3.1%)
Levofloxacin	14 (2.3%)	Levofloxacin	7 (2%)
Total Prescriptions	619 (100%)	Total prescriptions	358 (100%)

**Antibiotics prescribed at discharge:** At discharge, 321 (81.4%, n=394) cases had antibiotics prescribed for a mean duration of 5.7 ± 3.4 days. One antibiotic was prescribed for 284 (88.5%) cases, two for 35 (10.9%) cases and three antibiotics for two cases. The most common duration prescribed were 5 days (212, 66%), 7 days (85, 26.5%) and 3 days (13, 4%). Floxacillin (141, 43.9%), ampicillin-cloxacillin (63, 19.6%), metronidazole (45, 14%), cefuroxime (35, 10.9%), and amoxicillin-clavulanate (25, 7.8%) were the most common antibiotics prescribed (Table 1). The mean number of antibiotics prescribed was 1.1 ± 0.3 per patient. Twelve of the 30 patients without antibiotics during the course of admission had an antibiotic prescribed at discharge for a median duration of 5 days (range 1-7).

**Appropriateness of antibiotic prescription:** Evaluation of the treatment sheets revealed a high rate of documenting the route (363, 99.7%), frequency (362, 99.4%), and dose of antibiotics (363, 99.7%); the duration of antibiotics (40, 11%) was only rarely indicated. Only 29 (8%) patients had one or more antibiotics stopped, while 49 (13.5%) patients had one or more antibiotics added or changed. The proportion of prescriptions judged to be inappropriate was 45.4% (93, n=205) of antibiotics for prophylaxis, 33.4% (53, n=159) of antibiotics for treatment, 40.1% (146, n = 364) of antibiotics administered during hospitalization and 52.6% (169, n = 321) of antibiotics prescribed at discharge (Table 2). The most common reason for inappropriate antibiotic prescription during hospitalization was inappropriate duration (67/146, 45.9%), while at discharge it was inappropriate indication (155/167, 92.8%), as the patients were felt to have completed their antibiotics course and did not require further antibiotics

**Table 2:** Inappropriateness of antibiotics prescribed among inpatients at the surgical department between January and December 2015 at AIC Litein Hospital

Criteria	Prophylaxis (n=205)	Treatment (n=159)	Discharge (n=321)	Total
Inappropriate indication	39	4	155	198
Inappropriate choice	12	24	1	38
Inappropriate duration	42	25	11	77
Total	93 (45.4%)	53 (33.4%)	167 (52.6%)	313

## Discussion

In this audit, most (92%) patients admitted received one or more antibiotics, with the drugs being invariably (91%) administered intravenously (IV). In addition, the drugs were mostly continued till or just prior to discharge. The mean length of stay (7 days), differed from the mean duration of antibiotic administration by just one day. The percentage of patients receiving antibiotics for 1-7 days was similar to the percentage of patients whose length of stay was between 1 and 7 days, with only 8% of the patients having an antibiotic stopped prior to discharge. While prior studies have revealed a much a lower rate not only of total antibiotic administration (14-32%), but also proportion of IV formulation used (60-75%), we believe studies from other resource-limited settings may show a picture similar to ours [1,3,6]. This is because overuse of antibiotics is common in resource-limited facilities, with a preponderance for use of IV broad spectrum antibiotic formulations [3,4]. Severe or delayed disease presentation, inadequate use of bacterial culture and sensitivity studies, inadequate antimicrobial sensitivity resting capacity, higher patient expectation for antimicrobial therapy, absence of antibiotic prescription guidelines and psychological assumption of the superiority of IV antibiotics have all been stated to be reasons for the overuse of antibiotics [3,4,10]. Diagnostic uncertainty, low use of microbiology services, delay in release of antimicrobial susceptibility tests, poor compliance with guidelines, lack of awareness of international standards on antibiotic prescription have all been stated as reasons behind the predominant empirical antibiotic prescriptions [1,4,10-12]. Similar to Yilmaz et al., we noted 3rd generation cephalosporins, penicillins and metronidazole to be the most common antibiotics prescribed [2].

McCallum et al noted poor documentation of antibiotics prescribed with the stop dates and indication for the antibiotics indicated in 14% and 18% respectively [12]. Similarly, in this review, the duration of antibiotics was noted in only 11% of cases. Poor documentation leads to the unnecessary continuation of antibiotics as the clinicians lack adequate information to make an appropriate decision on whether to stop or continue or switch the antibiotics [9]. A well-documented antibiotic prescription should contain the indication, dose, clear date of administration, frequency, route and duration of administration. In addition, there is a need to review the clinical diagnosis and the need for continuing the antibiotics by 48 hours [9]. We believe the minimal or absent review of indication of antibiotics and poor documentation on the duration of the antibiotics on the treatment sheet led to the continuation of the antibiotics till discharge. This led prolonged antibiotic duration to be the most common reason (46% of cases) for irrational antibiotic use during hospitalization. Switch of antibiotics from intravenous to oral (IV-PO) can be undertaken in the vast majority patients, once clinical stability has been established and an equivalent or oral formulation is available, leading to improved patient satisfaction, reduced costs, reduced rate of complications, reduced time in preparation and drug administration, and earlier hospital discharge [12-14]. While only 13% of the cases had their antibiotic regimen altered, with some being switched to oral formulations, a more active IV-PO switch approach in this study would have led to a higher PO antibiotic use, and an earlier hospital discharge.

The task of documenting the discharge summary is commonly delegated to the most junior members of the team. In resource limited settings, however, there is significant variation in the training, competence and skill of the junior team members, who only passively participate in the overall patient management. There may be a strong desire on part of the team members to routinely send the patient home on a course of antibiotics, irrespective of the duration of antibiotics given while in the ward. In this review, the delegation or poor supervision of the medical staff (medical or clinical officer interns) writing the discharge summary and belief on routine prescription of antibiotics on discharge contributed greatly to the findings of 81% of patients being discharged on antibiotics, with 92% of these having a 5-7 day course. Interestingly, more than one-third of the patients who had not been on any antibiotic during their hospitalization were discharged with antibiotics for a median duration of 5 days. Antibiotic use can be improved by frequent audits, active surveillance, preparation of local antibiotic use guidelines, legislation about antibiotic restriction and initiation of antimicrobial stewardship programs [2,3,7,9]. The study had various limitations. The Interpretation of the appropriateness of the antibiotics may at times been biased by the quality of documentation and the opinions of the investigators. The retrospective nature of the review, lack of an infectious disease specialist in the team, and poor posting of the microbiology results in the charts were additional limitations.

## Conclusion

This audit found antibiotic prescription to be mostly inappropriate. Too many patients were placed on intravenous antibiotics that were continued for too long, with the patients then being discharged needlessly on more antibiotics. In addition, there was minimal review on the indication for and the need for an antibiotic switch or stop. Improved documentation, serial evaluation of the need for antibiotics, development of local antibiotic guidelines and better supervision of prescriptions at discharge may help improve the appropriateness of antibiotic use at AIC Litein Hospital.

### What is known about this topic

- Antibiotics are one of the most commonly prescribed medications in hospitalized patients, with a significant proportion used irrationally;
- The volume of antibiotic use is one of the most important factors driving the emergence of antibiotic resistance;
- Despite the dangers of antibiotic resistance, there continues to be misuse, overuse of antibiotics in many low and middle income countries where resources are limited.

### What this study adds

- Poor antibiotic prescription documentation and lack of frequent antibiotic prescription review leads to prolonged use;
- While irrational use of antibiotics within the hospital is important, this can continue when the patient is discharged on unnecessary antibiotics.

## Competing interests

The authors declare no competing interests.
